# Experiences of using a digital type 2 diabetes prevention application designed to support women with previous gestational diabetes

**DOI:** 10.1186/s12913-021-06791-9

**Published:** 2021-08-05

**Authors:** Winifred Ekezie, Helen Dallosso, Ponnusamy Saravanan, Kamlesh Khunti, Michelle Hadjiconstantinou

**Affiliations:** 1 Diabetes Research Centre, College of Life Sciences, University of Leicester, Leicester General Hospital, Leicester, LE5 4PW UK; 2 National Institute for Health Research (NIHR), Applied Research Collaboration (ARC) East Midlands, University of Leicester, Leicester, UK; 3 Leicester Diabetes Centre, University Hospitals of Leicester, Leicester General Hospital, Leicester, LE5 4PW UK; 4 Population, Evidence and Technologies, Division of Health Sciences, Warwick Medical School, University of Warwick, Coventry, UK; 5 Department of Diabetes, Endocrinology and Metabolism, George Eliot Hospital NHS Trust, Nuneaton, UK

**Keywords:** Gestational diabetes, Prevention, Type 2 diabetes, Qualitative research methods, Digital support, Mhealth

## Abstract

**Background:**

Gestational diabetes mellitus (GDM) is diagnosed during pregnancy, and women with a history of GDM are at a higher risk of developing type 2 diabetes mellitus (T2DM). Prevention strategies focused on lifestyle modification help to reduce long-term complications. Self-management technology-based interventions can support behaviour change and diabetes control. The Baby Steps programme, a randomised controlled trial intervention offering group education and access to a mobile web application, was evaluated to explore user experience of the app and barriers and facilitators to app usability.

**Methods:**

Ten semi-structured interviews and four focus group discussions were conducted with 23 trial participants between 2018 and 2019. Interviews and focus group discussions were audiotaped, transcribed and independently analysed. The analysis was informed by thematic analysis, with the use of the Nvivo 12 software.

**Results:**

Themes identified were: (1) GDM and post-pregnancy support from healthcare services; (2) Impact of Baby Steps app on lifestyle changes; (3) Facilitators and barriers to the usability of the Baby Steps app. The Baby Steps app served as a motivator for increasing self-management activities and a tool for monitoring progress. Peer support and increased awareness of GDM and T2DM enhanced engagement with the app, while poor awareness of all the components of the app and low technical skills contributed to low usability.

**Conclusions:**

This study documents experiences from existing GDM support, user experiences from using the Baby Steps app, and the barriers and facilitators to app usability. The app was both a motivational and a monitoring tool for GDM self-management and T2DM prevention. Peer support was a key trait for enhanced engagement, while barriers were low technical skills and poor awareness of the app components. A digital app, such as the Baby Steps app, could strengthen existing face-to-face support for the prevention of T2DM. The results also have wider implications for digital support technologies for all self-management interventions. Further research on the effect of specific components of apps will be required to better understand the long term impact of apps and digital interventions on self-management behaviours and outcomes.

**Trial registration:**

ISRCTN, ISRCTN17299860. Registered on 5 April 2017.

## Background

Gestational diabetes mellitus (GDM) is diagnosed in the second or third trimester of pregnancy [1], in line with the recent International Association of Diabetes and Pregnancy Study Groups Consensus Panel (IADPSG) diagnostic criteria. Women with a history of GDM are at least ten-fold higher risk of type 2 diabetes mellitus (T2DM), two-fold higher risk of cardiovascular disease and other long-term complications [2–7]. Prevention strategies focused on managing modifiable risk factors such as weight, physical activity, and dietary patterns can help reduce long-term complications [8]. Such lifestyle interventions have shown to be safe and cost-effective for reducing T2DM risks among those with GDM and pre-diabetes [9]; however, these have implementation implications, as they can be difficult to sustain and scale-up [10, 11]. Nevertheless, offering support, guidance, and strategies to reduce the risk of developing T2DM is recommended following a GDM diagnosis by the American Diabetes Association (ADA) and National Institute for Health Excellence (NICE) [1, 12].

Lifestyle modifications (diet and nutritional counselling, weight reduction and physical activity) and pharmacological interventions have been shown to reduce diabetes development [13]. The Diabetes Prevention Program for example, was a multicentre randomised clinical trial whereby women with and without previous history of GDM were randomised to metformin or intensive lifestyle intervention. Although metformin therapy appeared to be three times more effective in reducing the risk of diabetes in those with previous history of GDM compared to those without, it is important to note that both metformin and lifestyle interventions were shown to be equally effective interventions [14]. Lifestyle modifications alone have been found to be highly effective methods in preventing T2DM [15–17]. Self-management in particular is critical for T2DM prevention; and enhancing good self-care behaviours can be achieved through face-to-face and digital strategies [18, 19]. Digital technology provides easy on-demand access to information and social support which are needed by women with GDM who often have several time-demanding concerns [20]. These technologies have shifted management strategies from traditional face-to-face interventions toward interactive, self-directed, personalised, and cost-effective options; and, as a result, improved access to health care resources.

Self-management electronic and mobile health (eHealth and mHealth) technology-based interventions have shown positive impacts on behaviour change and diabetes control, including prevention of T2DM among women with GDM [21, 22]. These technologies employ a range of interactive behaviour change techniques and can be used continually over years [23]. MHealth applications (apps) provide options for personal tailoring of information, 24-h access to self-monitoring records, anonymity, motivation and social support networks [24]. Although mHealth services have positive impacts on health attitudes and behaviours among women with GDM [25, 26], only a few provide comprehensive, evidence-based educational content and tracking tools needed to monitor activities that reduce the risk of T2DM [27]. Consequently, most assessments of GDM digital interventions have mainly focused on clinical impact and usability, while information on user experiences related to functionality preferences, barriers and enablers to engagement is lacking [28, 29].

To establish whether digital self-management education programmes can improve physical activity levels in women with a previous diagnosis of GDM, we developed and tested an intervention (Baby Steps) in a parallel two-group randomised control trial (RCT) with women who had GDM during any pregnancy up to 60 months before the point of recruitment [30]. The overall aim of this diabetes prevention programme, was to improve lifestyle behaviours, in particular by walking and other physical activities.

### Baby steps intervention and app functionality

**Fig. 1:**
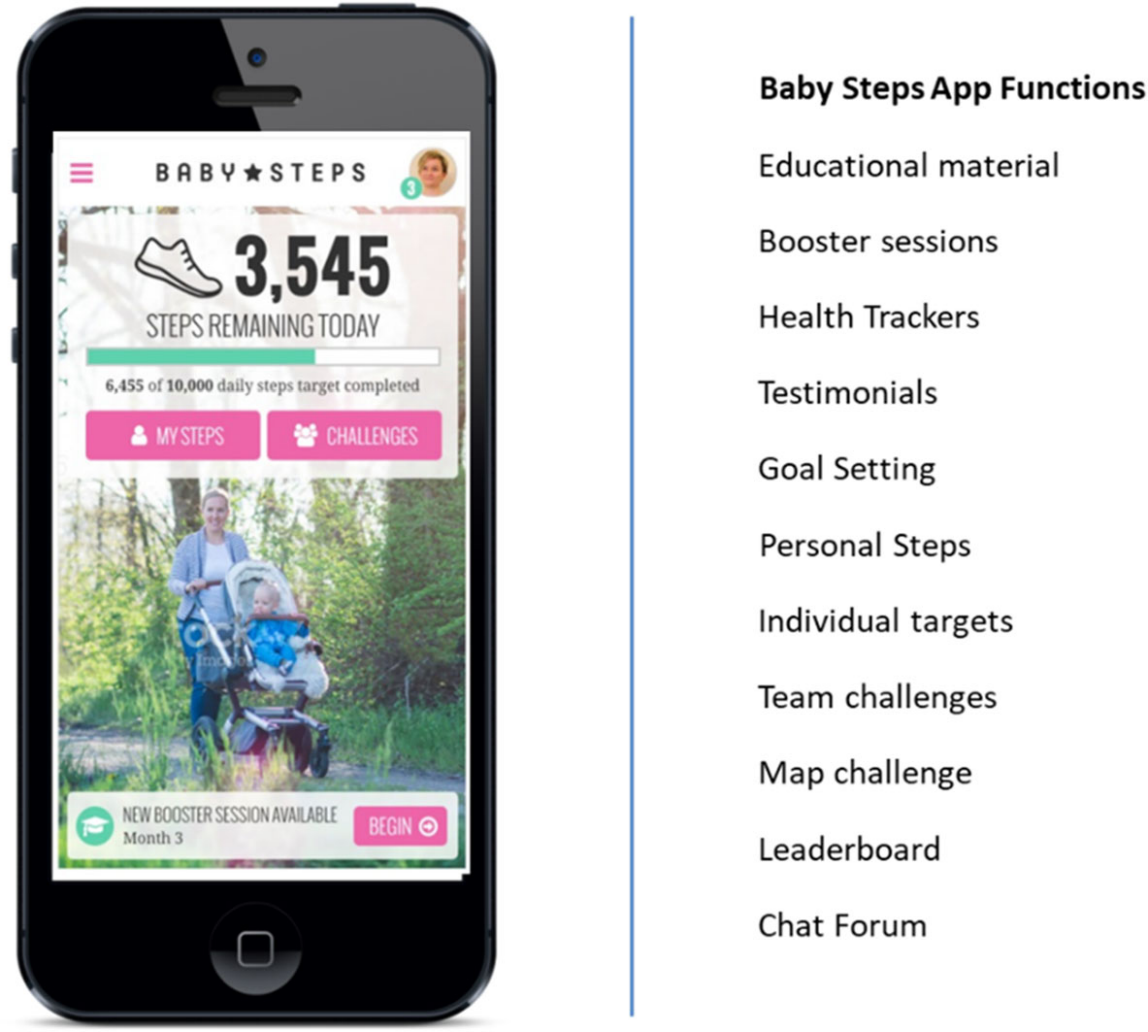
Baby Steps App components

The intervention comprised an evidence- and theory-based group education programme and access to a mobile web application (app) which provided an education component and interaction with a wrist-worn activity monitor given to the women during the group session. The app included the following functions: bite-sized interactive learning resources released in intervals, educational materials, chat forum, physical activity challenges (i.e. completing virtual map routes) linked with a leader board to allow participants to compete against each other; goal-setting to record personal goals, reminders to view new content or set new goals, and a function to download and track steps which can be connected to the mobile web app (Fig. 1). The research protocol and comprehensive details of the intervention design and study outcomes have been published [30–32].

### Aim

This qualitative study aimed to explore user experiences of the Baby Steps app and barriers and facilitators to app usability.

## Methods

### Study design and setting

This qualitative study was part of the Baby Steps study and recruited participants from both study sites in the United Kingdom; the University Hospitals of Leicester NHS Trust (serving the catchment area of Leicester City and the county of Leicestershire) and the George Eliot Hospital NHS Trust (serving the catchment area of North Warwickshire).

### Recruitment

Baby Steps participants were purposively sampled from the intervention arm when their participation in the RCT was complete. Recruitment was on the basis of app engagement during the trial, including regular and minimum users of the app. Although recruitment sample was based on this criteria, the aim of the analysis was not to compare these groups, but to rather capture a wide range of app usage and experience. Participants who had not attended the group programme or had withdrawn from the study were excluded.

#### Data collection

Focus groups were conducted at the Leicester Diabetes Centre, which lasted approximately 90 min. Telephone interviews were conducted for those who were unable to attend the focus groups and lasted approximately 30 min. The focus groups and interviews were carried out by our research team, including MH, who has extensive experience in qualitative research. A scriber supported the focus groups in case of any issues with audio recordings.

A topic guide for the focus group discussions was developed by our team to explore views about the Baby Steps app. This guide was refined for the telephone interviews to allow for further discussions about participants’ experience with GDM management. All participants provided written consent. Data were audio-recorded and transcribed verbatim by an independent professional transcriber.

#### Data analysis

Data were analysed using thematic analysis. Taking an inductive approach, data were initially coded and organised into framework matrices to identify patterns and enable comparison of codes. Data were subsequently coded into themes. This process was managed using Nvivo 12 software.

To ensure credibility, two independent researchers (WE and MH) coded and analysed the data (investigator triangulation). The two coders met regularly during the data analysis process, and any discrepancies were resolved through discussion, which led to a 100% agreement level.

## Results

### Participant characteristics

Ten telephone interviews (*n* = 10) and four focus groups (*n* = 13) were conducted between December 2018 and November 2019. Participants were aged between 25 and 50 years and were from different ethnic backgrounds, White British (*n* = 13), Indians (*n* = 5), Pakistani (n = 1), Arab (n = 1) and other Asian (*n* = 3), reflecting the Baby Steps study trial sample. Of these, 18 participants had registered and used the mobile web app in the RCT. Five participants had registered but not used the app. Both users and non-users were interviewed to explore facilitators and barriers to using a web application.

### Themes

The following three key themes were generated: (1) GDM and post-pregnancy support from healthcare services; (2) Impact of Baby Steps app on lifestyle changes; (3) Facilitators and barriers to usability of the Baby Steps app. Each theme is expanded into sub-themes for further exploration.

### GDM and post-pregnancy support from healthcare services

Participants shared their experience about receiving information at the time of GDM diagnosis by their healthcare providers. Many remembered receiving some type of information about GDM in the form of leaflets and booklets, which helped explain how to better understand and manage the condition:

*“I remember them getting a booklet, and then obviously they explained how you used the little thing that took your blood, what your readings had to be. And obviously, we were given information about certain foods that were obviously going to cause you more issues.”* (Participant 1, 45 years)

Despite the support that was provided to participants, some expressed disappointment about the lack of information and follow-up support provided at the time. For one particular participant, attending the Baby Steps programme helped her realise that she still remained at high risk of developing T2DM, even after her pregnancy, something that she was not made aware of previously:

*“I think I was just told that once you had the baby, you got rid of the diabetes and there hasn’t really been any follow-up. The only follow up was from this programme, which was amazing, because it obviously opened my eyes to the fact that I was at a higher risk of it (T2DM)”* (Participant 1, 45 years)

*“I got given a very sort of thin leaflet that basically gave …vague advice really. That was all I had…”* (Participant 9, 33 years)

When asked about the timing of information provision, participants felt that it was important that advice and support on GDM and the risk of T2DM, especially around blood sugar monitoring, should be made available and accessible not just during pregnancy but immediately at post-pregnancy also.

*“I felt that as soon as I had my little one, this sort of advice had stopped absolutely. I think I needed advice at the time… to continue monitoring my blood sugars after I had my son”* (Participant 8, 38 years)

In addition to information on GDM, participants were also asked whether information or support was provided specifically on making lifestyle changes to reduce the risk of T2DM, with many having no recollection of such support. Referring to food choices, one participant in particular “never knew that different foods would impact” on her. Personal experiences were shared around seeking lifestyle advice from friends:

*“With mine, I ended up going to my friend’s mum who’s insulin-dependent to find out most of the stuff… it was her that sat down and said ‘oh well you can't eat this, you can't eat that’ and explained it all to me”* (Participant 4, Focus Group 1)

*“I’ve got a friend who is a diabetic nurse as well, so I got some information from her”* (Participant 2, Focus Group 1)

Despite perceiving face-to-face support for self-management as helpful, participants seeked other avenues, especially when face-to-face support was scarce. Digital platforms were perceived as useful sources to seek advice and support on GDM and lifestyle management:

*“I’ll be honest, the information I was given from the hospital was quite poor, and I found myself doing a bit of research myself and found a really good group on Facebook and on-line actually.”* (Participant 7, 34 years)

### Impact of the baby steps app on lifestyle changes

The programme app provided participants with insight into their risk of developing T2DM. This included a deeper understanding of their family history, previous diagnosis of GDM and awareness to act soon to avoid developing T2DM in the future.

*“…I think suddenly all these things kind of came together, in alignment, made me realise I need to look after myself a lot better. And it’s been a really positive thing for me…”* (Participant 3, Focus Group 4)

*“…Knowledge given about how to change our lifestyle and the sessions really changed my life because I thought ‘right I need to lose weight, I need to do something to not develop this any further’ especially with my family history… the red light was just flashing.”* (Participant 1, Focus Group 4)

#### Increased physical activity and step count

During discussions with the participants, it was apparent that many were unaware of certain functions of the app, including the ‘booster sessions’ and ‘educational materials’. Nonetheless, for the functions that participants were aware of, the experience was described positively. Participants reported enjoying using the wrist-worn activity monitor that linked to the digital app. Being able to track their daily step count, kept them focused and encouraged them to meet the daily step count target over a long period of time.

*“I used the (wrist-worn activity monitor), it took me a little bit of time to get started on it, but I really, really enjoyed it. I really enjoyed using it, and I used it for a good few months actually because it kept me focused on making sure that I did 10,000 steps.”* (Participant 1, Focus Group 4)

Additional functions that participants accessed regularly were the ‘physical activity challenges’ and ‘leader board’ functions. One of the main purposes for these particular functions was to provide a platform for users to compete with each other. From the participants’ experiences, it was evident that competing and comparing themselves with others was a great boost to meet personal targets for some, but for others, this was less appealing.

*“…It was the competitive stuff, where you were comparing yourself against other people and other teams, and they could push me to do a lot more. I would do it, but it gave me that extra push to do a bit more just to beat them.”* (Participant 4, 39 years)

*“I didn’t like the competition part of it, sort of the leader board, because I thought as a group it’s, you know, we shouldn’t be against each other, which is why I liked the map really because it was a joint effort.”* (Participant 4, Focus Group 2)

Joining a virtual team or the global ‘chat forum’ where participants were able to share personal challenges and experiences with peers also helped increase their step count. The ‘chat forums’ appeared to serve as platforms for participants to track individual progress and keep motivated.

*“(The chat) part of the app I found that quite helpful just to keep motivated.”* (Participant 9, 33 years)

*“… Members on the forum are the same people who I met in group sessions here, so we got to know each other over a certain period of time, which was quite nice… you have that shared common interest, so we are all on the chat forums..”* (Participant 1, Focus Group 4)

Overall, the app was portrayed as a platform where participants felt encouraged to reflect on their current activity levels and change their behaviour accordingly.

*“I always used to think that the housework was sufficient, you’re doing so much housework you don’t really need to be so into exercise, but after using the wrist-worn activity tracker, you realise that, no, you know what, that’s not enough.”* (Participant 2, Focus Group 1)

#### Increased motivation

The Baby Steps app played an important role in keeping users motivated, whether this was with their steps, fitness, or overall health. One particular participant shared her excitement that even though she never perceived herself as ‘physically active’, she was still able to meet her target step goal set on the app.

*“I think it kind of, like with it counting your steps and uploading your steps onto it, what I did find was I was quite pleasantly surprised at the amount I was doing because I didn’t ever kind of count myself as physically active, but actually I was clocking up 18,000 steps without even trying.”* (Participant 9, 33 years)

The ‘physical activity challenge’ feature acted as a key motivator for participants to meet their step goals. They reported enjoying achieving their target and gaining rewards when they were at the top of their virtual league and leader board. Participants joined challenges to compete with others to achieve personal or group goals or merely enjoyed monitoring team progress on the ‘leader board’. Overall, this particular challenge and the opportunity to set group goals appeared to be a motivator on an individual and group level.

*“… it was good that you’re meeting your target and you are able to do more, you can even beat your target self… pushing yourself.”* (Participant 2, Focus Group 1)

*“I think having people in your team obviously motivates you to do more…if you had someone there doing it with you, then you wanted to do it as well so that you could keep up with them”* (Participant 1, Focus Group 1)

### Facilitators and barriers to usability of the baby steps app

#### Navigation system

The Baby Steps app was considered easy to use, but most found it challenging to navigate initially on their own. Some felt they were not ‘technically savvy’ enough. Others expressed frustration with the operation of the app, experiencing difficulties with the navigation system. These frustrations were partly due to the fact that participants were not fully aware of the available functions.

*“…There were a lot of things that I could have really used, a lot of things that were really good, but I didn’t know they were there, I didn’t know how to access them or anything and having somebody explain it and teach it to me better would have made a big difference to it.”* (Participant 4, Focus Group 1)

Suggestions were made for the provision of more technical guidance at the registration phase, with one-to-one support to ensure appropriate utilisation of the app features. Going through the app in a group setting was indicated to be beneficial, as participants were able to help each other log in and simultaneously get to know each other.

*“…We did it (went through the app) in our group when I was here for one of the sessions, so we all logged on at the same time, we all helped each other find the app and then we added each other while we were here so that we knew each other’s names.”* (Participant 1, Focus Group 1)

#### Peer support

Peer support was a key contributor to the usability of the app. The chat forum appeared to be a useful platform to elicit discussions and support amongst users. This type of communication was perceived as engaging by participants, emphasising the importance of peer interaction. Once the communication with others faded, some participants found it ‘pointless’ to continue with the forum.

*“I did read other people’s chats. I think that gave me some motivation…it’s still quite good to read what other people are thinking and doing.”* (Participant 3, Focus Group 1)

*“… From a team perspective, for the first few weeks, we were all like trying to talk to each other and help each other out, motivate each other, soon the team started losing people... it was difficult to be motivated…So it just became pointless.”* (Participant 6, 46 years)

Even though peer support was essential for some to continue with their personal progress, this was not a necessity for everyone. Some described peer support as a ‘socialising’ concept that did not suit everyone’s personality or needs. However, the idea of personalising this type of support by grouping participants with people they knew became appealing.

*“(Peer support) is socialising and I'm not a social person… I didn't like the idea of it being like a social thing…”* (Participant 4, Focus Group 1)

*“I would probably be a little bit apprehensive if it's just strangers, you know, typing 'I’ve done this, and I’ve done that’, well if it was a friend I’m doing it with, then I’ll probably find it a bit more easier to do. I think it’s a good idea.”* (Participant 1, Focus Group 3)

Having a group challenge forum with active participants was an important reason for many to continue using the app. Those who were part of less active groups indicated they often got frustrated since they had no one to motivate them or to work with directly with. In such cases, suggestions were made to extend the app to family or close friends. Some, in fact, extended their communication with fellow users by arranging to meet in person for walks.

*“…Some of them were meeting up with somebody else with their children in a pram and going for a walk, and I thought that was an idea.”* (Participant 3, Focus Group 1)

*“So having somebody else that was sort of going along the same as me, trying to eat healthier, trying to do more, it was really good because it felt like I had somebody to do it with.”* (Participant 8, 38 years)

## Discussion

This study aimed to explore user experiences of the Baby Steps app for women with previous GDM and the barriers and facilitators to the app’s usability. Overall, our findings suggest there is a strong need to provide follow-up support to women who have had GDM and who are at risk of T2DM, not just during pregnancy but also post-pregnancy. Our findings also suggest that a digital app, like the Baby Steps app, could be useful in supporting women in the post-GDM period as an educational and behaviour change tool to support key behavioural lifestyle changes. It is important when developing digital apps to include a ‘peer support’ element, as this was highlighted to be a key facilitator to the app usability.

Previous studies have shown women with GDM experience different challenges, including psychosocial factors such as stigma, abandonment and fear for their babies, which can be distressing to manage without adequate GDM information during pregnancy and after delivery, and without healthcare provider support [33–36]. Lack of awareness and understanding of GDM and the related impact also heightens the fears and risk of progression to T2DM [36, 37]. Therefore, knowledge of the risk factors that contribute to T2DM, as well as the awareness of living through GDM, is crucial for developing appropriate self-management and lifestyle changes. Providing information that is reliable and actionable through digital services, in addition to usual care, enhances diabetes self-management support. In addition to knowledge gaps, barriers to diabetes prevention and management are often related to insufficient time, reminders and support; hence, electronic apps are often preferred means of additional interventions [38–40]. This is shown in our study as the Baby Steps app provided both the essential information and support needed for T2DM prevention post GDM. Nevertheless, understanding the factors that influence the engagement pattern of users is essential for app development and usage [39, 41].

The major facilitator identified was the role of peer support as a motivator for active use of the app and for behavioural change. Social support has been shown to be a moderator for lifestyle changes, including physical activities [42]. Such support improves diabetes self-management both for those offering and receiving the support [43, 44]. Although peer support is not for all individuals, the social aspect of the Baby Steps app, including the chat forum and group challenges, appeared to encourage people to continue using the app, allowing them to exchange personal successes and challenges throughout their journey. In addition, the challenges for some were perceived as an opportunity to compete with each other, motivating them to meet their target goals and increase their steps. However, there is a group dynamic element in face-to-face group programmes that may not be possible to re-create on a digital platform. Reasons for not performing activities in groups included the apprehension of engaging with strangers and not having similar interests. Studies have also shown some women prefer to carry out certain activities in their own time and pace [20]. Hence, to harness the positive effects of group interactions, the familiarity between participants through meeting with other users, physically or virtually, and including options for setting up similar interest groups with self-paced activities should be encouraged [45]. Such peer support can relieve community health burdens, reduce costs and improve care access [46]. Therefore, personalisation options in apps, such as setting up interest groups, would address the individual factors that influence engagement. In addition, tracking and monitoring are key components to behaviour change [47, 48], and our study suggests that these components within the Baby Steps app could indeed promote behaviour change, since one advantage of self-monitoring is that participants are made more aware of their lifestyle, including diet and physical activities [20, 49]. Incorporating a peer support element with tracking in various functions (i.e. chat forum, and leader boards) could have a significant influence in one’s progress and outcomes [50]. Further research could explore the effect of each function.

Several eHealth interventions have been effective in affecting change in diabetes self-management behaviours. Key traits for developing adequate T2DM prevention digital support identified in this study include support after GDM diagnosis, accessibility to comprehensive digital resources, and social support. Motivation, however, was the central theme, and this included self- and group- motivation. Achieving personal milestones through group targets enhances motivation for healthy behaviour change [20]. Motivational facilitators are important for sustaining the healthy lifestyle needed to prevent T2DM, and apps often provide the motivation needed for behavioural modification and confidence in self-management among women with GDM [51, 52].

Barriers to using the Baby Steps app were generally associated with personal factors, technical knowledge and awareness of the contents of the app. These factors are often deterrents for engaging with T2DM prevention and management apps [39, 53–55]. Hence, digital interventions need to reflect participant preferences, technology usability, and face-to-face assistance [44, 56]. Participants’ unawareness of the full functionality of apps also presents a risk of misunderstanding digital intervention resources at an individual level which often lead to frustrations [51, 57]. As observed in other studies, this may also lead to a decline in engagement [44, 45]. In addition to these challenges, where physical interactions were lacking, participants sometimes sought guidance from other avenues. The availability of other support beyond the provided app interventions can help sustain activity and involvement [20]. Still, integrating face-to-face interactions (physical and virtual) and ongoing group support in apps could increase diabetes self-management and the success of digital interventions [58]. We would, however, emphasise that digital apps like Baby Steps are not to replace face-to-face programmes; preferably, they should be provided as alternative support formats.

Despite many participants being unclear about the functions of the programme and experiencing difficulties with the registration and navigation system, our study nonetheless highlighted the importance of such a programme to women with previous GDM. A digital app to support women who are at risk of developing T2DM could strengthen the existing face-to-face support provided by our healthcare services whilst improving psychosocial well-being. This holds true particularly during the current climate, whereby the COVID-19 pandemic has significantly restricted the provision of face-to-face T2DM prevention education programmes.

Guidelines recommend that women post-GDM should have regular screening for T2DM while being provided with lifestyle advice [12, 59, 60]. This includes mHealth and eHealth intervention, which have been shown to be cost-effective resources. Mhealth and eHealth digital support, such as the Baby Steps app, can help bridge the existing gaps in post-GDM care. Hence, harmonising similar initiatives into existing T2DM prevention programmes could support women in adopting and maintaining lifestyle changes to reduce future risk of T2DM. Therefore, the adjusted health service delivery structures present an opportunity to adapt to the current conditions and ensure that support can still be provided to those in need on a secure and user-friendly digital platform.

### Strengths and limitations

Our study included the use of an inductive thematic analysis methodology, which allowed for a broader exploration of participants’ experiences. To establish trustworthiness, our study met the following criteria for methodological rigour: credibility (researcher triangulation, detailed description of data, member checking and debriefing of data coding and report), dependability (data was audio recorded and transcribed by an independent transcriber), confirmability (applied reflexivity during data collection and analysis, by involving more than just one researcher during the research process to avoid researcher bias) and transferability (detailed information about study methods and participants).

The findings add to the literature regarding digital support interventions for the prevention of T2DM among women with a history of GDM. Although focus groups can be a method to encourage and elicit group discussion, we should acknowledge that such methods may also limit diverse experiences among participants with different views. Having said that, the addition of one-to-one telephone interviews and the combination of the two modes of data collection would have reduced this limitation. Our participants spanned a wide range of ages and ethnic backgrounds. As the trial was restricted to women who spoke English, this will have limited our ability to meet all language and cultural needs. We interviewed more people in Leicester compared to Nuneaton, which may be seen as a limitation; however, the purpose of this qualitative study was to explore experiences of those using the app, not to compare users across sites. Due to the small sample size, the group interviewed may not necessarily represent the experiences of all study participants. Moreover, participants who had withdrawn from the study trial were excluded from this qualitative study, potentially limiting our understanding about other barriers for engaging with the Baby Steps app. Therefore, future research could explore the target population (including those who do not attend intervention activities) and further explore age and ethnic influence on engagement with T2DM prevention digital supports. The researcher who conducted the focus groups was involved in the data analysis, which may lead to bias. However, to reduce this bias, a scribe supported the focus groups and a second coder helped with the analysis.

## Conclusions

Understanding the barriers and enablers to app usability is important for developing and implementing motivational digital programmes for T2D prevention. The Baby Steps app was reported to be both a motivational and monitoring tool to adopt and maintain lifestyle changes, with the ‘peer support’ featuring as a key contributor to the usability of the app. Digital programmes could become a useful additional resource to strengthen the existing face-to-face support provided by healthcare services to prevent T2DM among women with post GDM.

## Data Availability

The data that support the findings of this study are available from the Leicester Diabetes Centre but restrictions apply to the availability of these data, which were used under license for the current study, and so are not publicly available. Data are however available from the authors upon reasonable request and with permission of the Leicester Diabetes Centre.

## Abbreviations

T2DM: Type 2 diabetes mellitus
GDM: Gestational diabetes mellitus
RCT: Randomised controlled trial
